# Emerging Roles of *Ganoderma Lucidum* in Anti-Aging

**DOI:** 10.14336/AD.2017.0410

**Published:** 2017-12-01

**Authors:** Jue Wang, Bin Cao, Haiping Zhao, Juan Feng

**Affiliations:** ^1^Department of Neurology, Shengjing Hospital, China Medical University, Shenyang, 110004, China; ^2^Cerebrovascular Diseases Research Institute, Xuanwu Hospital of Capital Medical University, Beijing, 100053, China

**Keywords:** *Ganoderma lucidum*, anti-aging, antioxidant, immunomodulation, anti-neurodegeneration

## Abstract

*Ganoderma lucidum* is a white-rot fungus that has been viewed as a traditional Chinese tonic for promoting health and longevity. It has been revealed that several extractions from *Ganoderma lucidum*, such as Ethanol extract, aqueous extract, mycelia extract, water soluble extract of the culture medium of *Ganoderma lucidum* mycelia, Ganodermasides A, B, C, D, and some bioactive components of *Ganoderma lucidum*, including Reishi Polysaccharide Fraction 3, *Ganoderma lucidum* polysaccharides I, II, III, IV, *Ganoderma lucidum* peptide, *Ganoderma* polysaccharide peptide, total *G. lucidum* triterpenes and Ganoderic acid C1 could exert lifespan elongation or related activities. Although the use of *Ganoderma lucidum* as an elixir has been around for thousands of years, studies revealing its effect of lifespan extension are only the tip of the iceberg. Besides which, the kinds of extractions or components being comfrimed to be anti-aging are too few compared with the large amounts of *Ganoderma lucidum* extractions or constituients being discovered. This review aims to lay the ground for fully elucidating the potential mechanisms of *Ganoderma lucidum* underlying anti-aging effect and its clinical application.

## 1. Introduction

*Ganoderma lucidum* (Fr.) P. Karst is a basidiomycete white rot fungus commonly known as “Ling Zhi” in China, “Rei Shi” in Japan and “Youngzhi” in Korea. The pharmacological effect of *Ganoderma lucidum* was first attested by “Shen Nong’s Herbal Classic” as early as 100 BC, and observed to promote health, increase vigor and vitality as well as prolong lifespan [1]. The ancient Chinese Taoist viewed *Ganoderma lucidum* as an herbal medicine that could help people to achieve the “elixir of external youth”. In China, *Ganoderma lucidum* has long been used as a folk medicine for improving health and is considered the most exalted traditional Chinese medicine [2].

Analysis of non-volatile ingredients in *Ganoderma lucidum* showed that it contains 1.8% ash, 26-28% carbohydrate, 3-5% fat, 59% fiber and 7-8% protein [2]. The main active constituents, including polysaccharides, triterpenes and peptidoglycans, are found in the fruit body, mycelium and spore [3]. Beseids which, there are many different extractions of *Ganoderma lucidum* due to the specific extracting procedures used during production and the part of plant it gets from. Regarding to the anti-aging and related functions of *Ganoderma lucidum,* the main *Ganoderma lucidum* extractions are ethanol extract, aqueous extract of *Ganoderma lucidum* and the extract from the mycelia and spores of *Ganoderma lucidum.* The bioactive components of *Ganoderma lucidum* with anti-aging or anti-aging related functions meanly includes polysaccharides, triterpenes and peptides.

Aging is almost always accompanied by a decline in bodily physiological function, resulting in an increased susceptibility to age-related disorders. It is an inevitable physiological process, but the underlying mechanisms remain to be elucidated after many decades. Among the numerous theories associated with aging, the oxidative stress and free radical accumulation theories stand out the most. The antioxidant system deteriorates as a function of age, bringing about disruption of the delicate balance between radical oxygen species production and elimination leading to oxidative cellular damage [4]. Post-mitotic tissues such as the brain, heart and skeleton muscle are more susceptible to aging, compared with other organs [5]. Importantly, oxidative stress accumulation and mitochondrial dysfunction are important inducers of cardiac aging [6]. Cardiac contraction is dependent on oxidative phosphorylation (OXPHOS) and the mitochondrial electron transport chain (ETC). Their dysfunction may increase ROS production to an unhealthy level, thereby giving rise to structural and functional changes in the myocardium, such as myocardial atrophy or compensatory hypertrophy, which induces cardiac aging [7]. In the brain, the accumulation of free radicals and attenuation of respiratory chain enzyme complex activity cause damage to cerebral mitochondria [8], wherein their dysfunction can induce the onset of some neurodegenerative diseases, such as Parkinson’s disease, Alzheimer’s disease, Huntington’s disease, among others.

**Table 1:** The origin, function and mechanisms of *Ganoderma lucidum* extracts in anti-aging or anti-aging related effects.

Extraction	Origin	Function	Mechanisms	Refs.
Ethanol extract of *Ganoderma lucidum* (EGL)	Fruit body	Lifespan elongation activity	Inhibit ROS production, lipid peroxidation, advanced oxidation protein products; Increase production of mitochondrial electron transport complexes, Mn-SOD, CAT, GSH and GSH-Px, DPPH and ABTS radical scavenger activities and FRAP	[20-24, 26]
		Immunomodulatory effect	Increase expressions of TLR4 and MyD88	[28]
		Antioxidant activity	Increase expression and phosphorylation of Nrf2 to induce the upregulation of HO-1	[29, 34]
*Ganoderma lucidum* aqueous extract (GLA)	Fruit body	Antioxidant activity	Increase radical scavenging activity and ferric reducing antioxidant power	[38]
		Anti-neurodegeneration	Inhibit synaptophysin transportation, JNK and p38 signaling pathway to antagonize neuronal apoptosis	[39]
*Ganoderma lucidum* mycelia extract	Mycelia	Neuronal differentiation promoting effect	Induce Erk1/2 and CREB phosphorylation Increase the secretion of non-amyloidogenic protein secretion (sAPPα) and expression of the amyloid precursor protein (APP)	[40, 41, 43]
Water soluble extract of the culture medium of *Ganoderma lucidum* mycelia (MAK)	Mycelia	Antioxidant activity	Inhibit lipid peroxidation and ROS production Increase SOD, CAT and GSH productions	[47-49]
Ganodermasides A, B, C and D	Spores	Lifespan elongation activity	Increase expression of Skn7 to induce production of UTH1	[50]

ROS: reactive oxygen species, SOD: superoxide dismutase, CAT: catalase, GSH: glutathione, GSH-Px: glutathione peroxidase, DPPH: 2,2-diphenyl-1-picrylhydrazil, ABTS: 2,2’-azinobis (3-ethylbenzothiazolin-6-sulphonic acid), FRAP: ferric reducing antioxidant power, TLR4: toll-like receptor 4.

Besides oxidative stress, aging is also closely associated with bringing about structural and functional defects in the immune system [9]. Immunological dysfunction could be the cause of the increased susceptibility of the aged population to bacterial and virus infections, which are commonly seen in the elderly.

Gradual loss of cognition is one of the main characteristics of aging, with manifestation of declining logical thinking, memory and spatial abilities. Cerebral aging is the main cause of cognitive deficits and could be induced by neurodegeneration [10]. While, on the other hand, age-associated cognitive deficits do not mean neurodegenerative diseases such as Alzheimer’s disease or Parkinson’s disease [12], since aging brain applies a sensitive microenvironment to induce more severe damage than that caused by diseases [13]. The demise of neurons caused by the activation of cell death programs is involved in the process of age-related neurodegeneration [14].

Although *Ganoderma lucidum* has been used as an elixir for thousands of years, studies revealing its anti-aging effect and lifespan extension are only the tip of the iceberg. Whether *Ganoderma lucidum* exerts an anti-aging effect remains a mystery. Therefore, this review aims to lay the ground for fully elucidating the potential mechanisms of *Ganoderma lucidum* underlying anti-aging effect to promote its clinical application as an anti-aging herbal medicine.

## 2. Anti-aging and anti-aging related effects of *Ganoderma lucidum* extractions

The extractions of *Ganoderma lucidum* with direct lifespan elongation effects or potential anti-aging properties mainly includes the Ethanolic extract of *Ganoderma lucidum* (EGL), *Ganoderma lucidum* aqueous extract (GLA), *Ganoderma lucidum* mycelia extract, Water soluble extract of the culture medium of *Ganoderma lucidum* mycelia (MAK) and Ganodermasides A, B, C and D. These extracts are obtained from different parts of *Ganoderma lucidum.* Their origin, function, and mechanisms are shown in Table 1.

### 2.1 Extracts obtained from the fruit bodies of *Ganoderma lucidum*

#### 2.1.1 Ethanol extract of *Ganoderma lucidum* (EGL)

EGL is extracted from the fruit body of *Ganoderma lucidum* with 25% ethanol [15]. Its anti-aging effect has been proven in animals. EGL also has several anti-aging related effects, which supports its clinical application as an anti-aging drug.

##### 2.1.1.1 EGL-mediated lifespan extension

The mitochondria play an important role in the aging process since it produces ATP and exerts a protective effect against aging by increasing basal metabolic rate or inhibiting senescent accelerating processes. Aging causes mitochondrial dysfunction, excessive ROS production and decreases the activities of antioxidant enzymes [16]. ROS overload leads to oxidative modification of various enzymes causing changes in their structures and decrease in enzymatic activities [17]. Furthermore, the decreased activities of antioxidant enzymes in the aged population is also associated with mitochondrial transcription and translation disorder, which is caused by age-related ROS accumulation resulting in mitochondrial DNA mutation [18]. EGL could protect the heart, liver and brain against aging in mice mainly through its antioxidant effect. The use of EGL in aged mice has been shown to decrease the level of ROS to delay the aging process [19].

The dysfunction of antioxidant enzymes accelerates the aging process as they are the first line of defense for protecting biological macromolecules against oxidative stress. EGL could increase the activity of antioxidant enzymes including glutathione peroxidase (GPx), catalase (CAT) and manganese-superoxide dismutase (MnSOD) in the cardiac, hepatic and cerebral mitochondria of aged mice. Glutathione (GSH) is the richest non-protein thiol molecule in tissues and possesses the ability to prevent cerebral ROS accumulation [20] through a direct reaction with ROS and electrophilic metabolites. The aging process gives rise to excessive lipid peroxidation (LPO) products such as malonaldehyde (MDA), 4-hydroxynonenal and ROS, which could accelerate the consumption and degradation of GSH. EGL reverses the decrease of GSH in aged mice. Furthermore, the production of free radicals during the process of senescence induces the accumulation of LPO products, which is viewed as a decrease in the protection of enzymatic or non-enzymatic antioxidant substances [21]. It has been confirmed that aging induces the increase in the level of oxidative protein [22] and advanced oxidation protein products (AOPP) is a type of dityrosine that contains cross-linked protein products, reflecting the degree of protein damage induced by oxidative stress [23]. The main mechanism of protein peroxidation lies in the formation of ^?^OH from iron located in the mitochondrial membrane through Fenton’s reaction. Since EGL is equipped with the ability of increasing the level of antioxidant enzymes and GSH, it could decrease the level of LPO in aged mice. Besides which EGL reverses the upregulation of AOPP in aged mice. EGL also possesses robust free radical scavenging ability catalyzed by 2,2-diphenyl-1-picrylhydrazil (DPPH), 2,2’-azinobis (3-ethylbenzothiazolin-6-sulphonic acid) (ABTS) and ferric reducing antioxidant power (FRAP) [24].

α-ketoglutarate dehydrogenase (α-KGDH) and pyruvate dehydrogenase (PDH) are two tricarboxylic acid cycle dehydrogenases that are positively associated with the level of NADPH; they promote the formation of the mitochondria electron transport complex I. It has been confirmed that in multiple brain regions of aged mice, the levels of α-KGDH and PDH are decreased due to free radical attack [25]. The use of EGL in aged mice significantly increases the levels of cerebral mitochondrial α-KGDH, PDH and respiratory chain complex I. Furthermore, EGL could increase mitochondria electron transport complex II activity in the brain by augmenting the level of succinate dehydrogenase (SDH). Besides the brain, the heart is another organ susceptible to the injury induced by the aging process. EGL could increase the activities of tricarboxylic acid cycle dehydrogenases (MDH, SDH and α-KGDH) and cardiac mitochondria electron transport complexes I, II and IV in aging heart [26].

##### 2.1.1.2 The anti-aging related functions of EGL

###### Imunomodulatory and anti-neurodegeneration effects of EGL

The immunomodulatory effect of EGL mainly manifests as the inhibition of cytokine and inflammatory mediators (COX-2, iNOS, NO, TNF-α, IL-1β) released by microglia upon LPS stimulation. These inflammatory mediators and cytokines have close relationships with neurodegeneration. For example, the inhibition of COX-2 could relieve cerebral ischemic injury and slow down the progress of Alzheimer’s disease or Parkinson’s disease [27]. The release of iNOS and TNF-α by microglia during neurodegeneration could accelerate the process itself. Moreover, IL-1β acts on microglia or neurons through the IL-1 receptor and is directly related to neurodegeneration. All these facts provide strong evidence for the treatment of age-related neurodegenerative diseases by EGL.

The Toll-like receptor (TLR) is a family of receptors associated with the innate immune system. When TLR is activated by some specific extracellular stimulation, its intracellular domain would combine with MyD88 and this process activates key inflammatory transcription factors, such as NF-κB, to induce an inflammatory reaction. Upon LPS stimulation, EGL could inhibit the expressions of TLR4 and MyD88 and decrease the level of downstream inflammatory activation [28].

###### Antioxidant effect of EGL

EGL exhibits antioxidant effects under normal circumstance apart from aging. Nrf2 is a leucine zipper transcription factor that could perceive the onset of intracellular oxidative stress and react quickly to any changes in the cell’s redox state [29]. Nrf2 stably binds with Kelch-like ECH-associated protein 1 under normal condition to form Nrf2/Keap1 complex. Phase II enzyme could lead to the dissolve of Nrf2/Keap1 complex, then the free Nrf2 could translocate to nucleus [30]. Nrf2 could bind to and activate the antioxidant response element (ARE) in the nucleus to maintain intracellular redox homeostasis [31]. Heme oxygenase-1 (HO-1) is the downstream effector of the Nrf2-ARE pathway. HO-1 could catalyze heme to form carbon monoxide, billiverdin and ferrous iron to neutralize intracellular ROS [32] and protect the cell against oxidative injury [33]. EGL has been shown to increase the expression and phosphorylation of Nrf2 that leads to the upregulation of HO-1 to exert its antioxidant effect [34].

###### Anti-tumor effect of EGL

EGL has pro-apoptosis, anti-proliferation and anti-angiogenic effects, which makes it an ideal tumor combating drug. EGL could induce apoptosis in human breast cancer cells by altering mitochondrial transmembrane depolarization and exerting its anti-proliferative effect through cell cycle arrest [35].

###### Protective effect of EGL against male lower urinary tract symptoms

It has been demonstrated in a randomized clinical trial that the use of EGL in a group of males over the age of 49 with slight to moderate lower urinary tract symptoms for 12 weeks could drastically improve their International Prostate Symptom Score, suggesting that EGL is effective in the treatment of lower urinary tract symptoms [36].

#### 2.1.2 Aqueous extract of *Ganoderma lucidum*

The *Ganoderma lucidum* aqueous extract (GLA) can be prepared by dehydrating the air-dried *Ganoderma lucidum* fruit bodies (1kg) with 95% ethanol then boiling the powder in 20L water for 6h and subsequently filtering the liquid extract with trichloroacetic acid to remove protein ingredients. GLA is mainly composed of carbohydrate and protein other than triterpenes [37]. As a common extract of *Ganoderma lucidum*, GLA has been reported to possess many beneficial effects such as anti-tumor, anti-diabetic, hepato-protection and many others. Regarding its potential application as an anti-aging drug, we will mainly discuss its antioxidant and anti-neurodegeneration properties, keeping in line with the aim of this review.

##### 2.1.2.1 Antioxidant property of GLA

It has been reported that GLA possesses an antioxidant property manifesting as strong radical scavenging activity, indicating its suitability as an anti-aging drug. However, the antioxidant activity of GLA is weaker compared with the methanolic extract of *Ganoderma lucidum* as the antioxidant activity is positively correlated with phenol content and the amount of phenol content in the methanolic extract is much higher *vs.* GLA [38].

##### 2.1.2.2 Anti-neurodegenerative effect of GLA

Another evidence for the potential anti-aging effect of GLA lies in its property of protecting against age-related neurodegenerative diseases. AD is one of the most serious neurodegenerative diseases and GLA has been proven to be effective in the treatment of AD in a cellular model of primary cultured neurons treated with the Aβ peptide. AD causes synaptic degeneration, loss of synapses, decrease in the immunoreactivity of the synaptic marker-- synaptophysin and induces its accumulation. The use of GLA reverses the blockage of synaptophysin transport [39]. Further, GLA significantly inhibits JNK and the P38 signaling pathway to antagonize neuronal apoptosis induced by Aβ peptide [39]. Although the effect of GLA in treating other age-related neurodegenerative diseases has never been tested, it deserves further study since the pathophysiological process of AD and the other age-induced neurodegeneration are similar.

### 2.2 Extractions from mycelia of *Ganoderma lucidum*

#### 2.2.1 *Ganoderma lucidum* mycelia extract

Many nutrients in the fruit bodies of *Ganoderma lucidum* are produced in the mycelium, indicating the critical role it plays in the production of bioactive substances. The *Ganoderma lucidum* mycelia extract is prepared as follows: *Ganoderma lucidum* is cultured in a potato dextrose broth at 28? for 28 days to obtain the mycelia. The product is degreased using 95% ethanol then autoclaved at 121? for 20 min. The final extract is composed of 69.4% carbohydrate and 19.9% protein.

The potential anti-aging effect of the *Ganoderma lucidum* mycelia extract is exerted through the promotion of neuronal differentiation [40]. Nerve growth factor (NGF) withdrawal during culturing of PC12 cells causes apoptosis while the corporation of *Ganoderma lucidum* mycelia extract could induce the differentiation of PC12 cells and prevent NGF-withdrawal-induced PC12 cell apoptosis, suggesting that there exist neuroactive compounds in *Ganoderma lucidum*, which harbor potential anti-neurodegenerative effects [41]. Furthermore, *Ganoderma* mycelia extract could induce ERK1/2 and CREB phosphorylation [41], wherein CREB phosphorylation has been demonstrated to be associated with learning, memory and long term potentiation in the hippocampus [42]. This extract has also been shown to increase the secretion of non-amyloidogenic protein secretion (sAPPα) and the expression of amyloid precursor protein (APP) in SH-SY5Y cells [43]. APP and sAPPα have neurotropic and neuroprotective effects that promote the formation and reparation of synapses [44] as well as drive the differentiation of human myeloid progenitor cells to a neuronal phenotype [45]. The extracts induce the secretion of sAPPα mainly through the activation of ERK1/2 and PKC and subsequently, their complex downstream signaling cascades [43].

#### 2.2.2 Water soluble extract of the MAK

MAK is the water-soluble extract of the culture medium of *Ganoderma lucidum* mycelia, which consists of multiple ingredients similar to the fruit body of *Ganoderma lucidum* such as polysaccharides, glucans and triterpenes. Polysaccharides and other nutritional ingredients could be secreted into the culture medium during the growth of *Ganoderma lucidum* mycelia, so MAK may have similar effects as *Ganoderma lucidum* mycelia extract [46]. MAK is obtained by culturing *Ganoderma lucidum* in a solid culture medium for about 3 to 4 months until the time just before the formation of the fruit body. The entire medium is then extracted with hot water followed by filtration and lyophilization of the solution to acquire the extract in a powdered form [46].

MAK possesses radical-scavenging ability and suppresses lipid peroxidation in a concentration dependent manner [47]. Oral administration of MAK for 2 weeks could protect diabetic rat brains from oxidative stress through the decreased ROS production and increased levels of antioxidant enzymes, including superoxide dismutase, catalase and glutathione peroxidase [48]. Furthermore, MAK could decrease the oxidative stress induced by cerebral ischemia reperfusion to exert neuroprotection [49]. Whether MAK possess anti-aging capabilities remains unclear, further research is needed to properly elucidate the components in MAK and whether they possess anti-aging effect.

### 2.3 Extractions from spores of *Ganoderma lucidum* - Ganodermasides A, B, C and D

**Figure 1.**
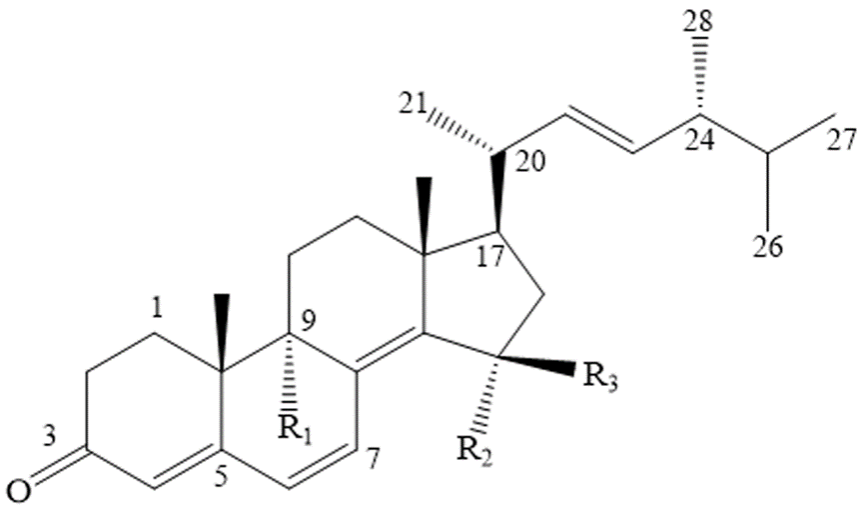
The structure of Ganodermasides A, B, C and D. 1 represents A, 2 represents B, 3 represents C, 4 represents D; 1: R_1_=H, R_2_=OH, R_3_=H; 2: R_1_=H, R_2_=H, R_3_=OH; 3: R_1_=OH, R_2_, R_3_=O; 4: R_1_=OH, R_2_=H, R_3_=H.

Since the spores of *Ganoderma lucidum* are difficult to collect, which makes it difficult to study their function. Therefore, the study of *Ganoderma lucidum* spores is commonly focused on the extraction and function of its fruit body. Thankfully, the recent successful indoor cultivation of *Ganoderma lucidum* spores makes it possible to collect them. Ganodermasides A, B, C and D are ergosterol derivatives isolated from the methanol extract from spores of *Ganoderma lucidum*. The structures of Ganodermasides A, B, C and D are shown in Figure 1.

Coincidently, all the four active compounds share similar mechanisms of lifespan extension tested in yeast [50]. UTH1 has been identified as an age-related gene in yeast, which participates in stress defense and lifespan elongation [51]. Skn7 is a transcription factor that regulates UTH1 expression. It has been demonstrated that Ganodermasides A, B, C and D extend yeast lifespan through UTH1, which is regulated by Skn7 [50].

## 3. Anti-aging and anti-aging related effects of bioactive components in *Ganoderma lucidum*

The bioactive components in *Ganoderma lucidum* that mediate their anti-aging or anti-aging related functions mainly include polysaccharides, triterpenes, peptides and polysaccharide peptides. We will first outline their general characteristics then describe their lifespan extention and related effects.

Polysaccharides are the most important contributors to the bioactivity and medical application of *Ganoderma lucidum*. It has been reported that these polysaccharides are equipped with antioxidant and immunomodulatory properties. Considering the structure of the polysaccharides extracted from *Ganoderma lucidum*, they are made up of glucose, mannose, galactose, fucose, xylose and arabinose. Even different types of *Ganoderma lucidum* share a distinct glycosidic linkage, giving the polysaccharides the opportunity to bind with various protein or peptide residues to form polysaccharide-protein or -peptide complexes. Based on the distinct structure and water solubility of polysaccharides, their extraction pattern differs from each other. Generally, the extracting processes include crude extraction, precipitation and purification. The most common methodology for primary extraction is hot water extraction with other techniques involving microwave, ultrasonic, ultrasonic/microwave and enzymatic treatments. The crude solution is then precipitated by the addition of alcohol, methanol or acetone. Finally, the purification process (by chromatographic techniques, such as ion-exchange, gel filtration and affinity chromatography) aids in obtaining pure polysaccharides.

**Figure 2.**
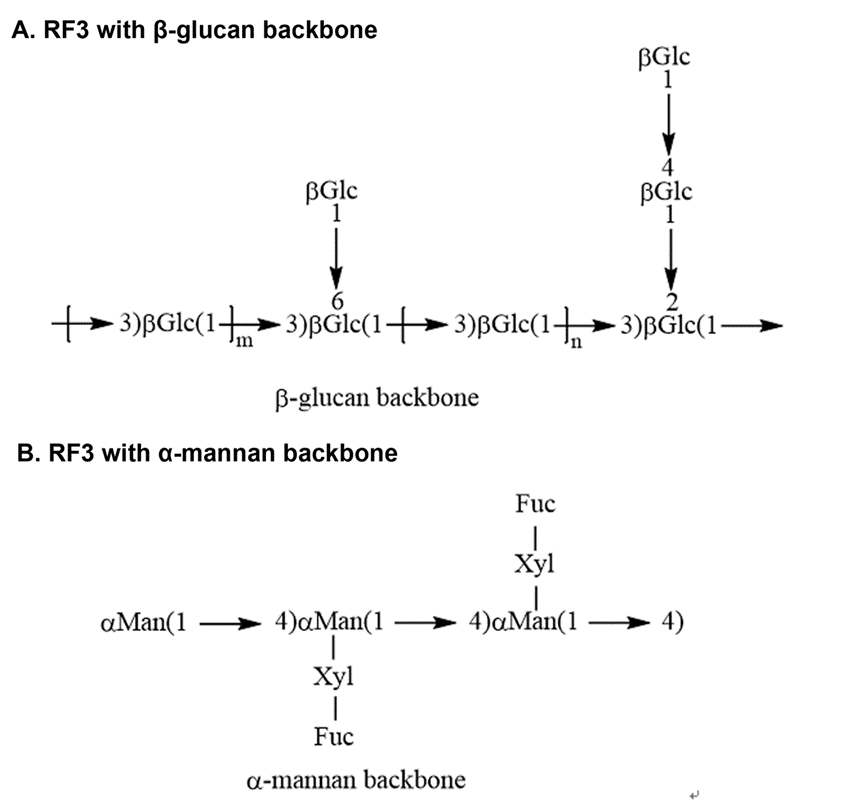
The structure of Reishi Polysaccharide fraction 3 (RF3) with different glycol backbone **A**) The structure of RF3 with β-glucan backbone. **B**) The structure of RF3 with α-mannan backbone.

Studies have revealed that the polysaccharides in *Ganoderma lucidum* possessing prominent lifespan extension effects mainly include the Reishi Polysaccharide Fraction 3 (RF3) and *Ganoderma lucidum* polysaccharides I, II, III and IV (GLPI, GLPII, GLPIII and GLPIV). RF3 is a water-soluble glycol-conjugate fraction of *Ganoderma lucidum* while *Ganoderma lucidum* polysaccharides I, II, III and IV (GLPI, GLPII, GLPIII and GLPIV) are extracted from *Ganoderma lucidum* mycelium using fermented soybean curd residue as a growth medium, which is a new technique for extracting *Ganoderma lucidum* polysaccharides. The traditional production of *Ganoderma lucidum* polysaccharides is mostly extracted from *G. lucidum* fruit bodies, which require a long incubation time with low yield. The new technique used in extracting GLPI, GLPII, GLPIII and GLPIV not only saves cuts down production time of *Ganoderma lucidum* polysaccharides, but also increases the utilization of agricultural waste and subsequently decrease the cost of extraction [52]. RF3, GLPI, GLPII, GLPIII and GLPIV have all been reported to have immunomodulatory effects. RF3 have also demonstrated certain anti-aging effects while GLPI, GLPII, GLPIII and GLPIV may induce lifespan extension through their antioxidant properties [53]. The structure of RF3 is shown in Figure 2 and the monosaccharide composition of the four polysaccharides are shown in Table 2.

**Table 2:** Monosaccharide composition of GLPI, GLPII, GLPIII and GLPIV.

Sample	Composition	Molar ratio
GLPI	Ara, Rha, Xyl, Man, Glu	4.66: 1.23: 3.14: 0.61: 1.29
GLPII	Ara, Xyl, Glu	2.82: 1.33: 0.87
GLPIII	Ara, Rha, Xyl, Gal, Man, Glu	5.09: 0.52: 1.07: 1.29: 0.48: 2.76
GLPIV	Ara, Rha, Fuc, Xyl, Man, Glu	4.73: 0.65: 0.72: 2.27: 0.52: 0.92

Ara:Arabinose,Rha: rhamaose, Xyl: xylose, Man: mannose, Glu: glucose, Gal: galactose,Fuc: fructose.

Bioactive peptides are a class of peptides with molecular masses of less than 6 kDa, the relatively small molecular weight enables it to be easily absorbed by the human intestine [54]. *Ganoderma lucidum* peptide (GLP) is the bioactive peptide of *Ganoderma lucidum*. It has been reported that GLP is the major antioxidant component of *Ganoderma lucidum*, and this property makes it a probable candidate of anti-aging.

Since the individual polysaccharides and peptides from *Ganoderma lucidum* are candidates for mediating its anti-aging effect, the combination of these components may also have similar functions. The *Ganoderma* polysaccharide peptide (GLPP) has been shown to possess antioxidant and immunomodulatory effects, thus having the potential of being exerting an anti-aging effect. Its average molecular weight is 520 kDa. The polysaccharides in GLPP are D-rhamnose, D-xylose, D-fructose, D-galactose, D-glucose with molar ratios of 0.549: 3.614: 3.167: 0.556: 6.89 and are linked by β-glycosidic linkages. GLPP contains 16 amino acids, Asp 8.49, Thr 3.58, Ser 3.93, Glu 5.81, Gly 3.50, Ala 3.84, Cys1.06, Val 2.68, Met 5.33, Iso-Leu 0.25, Leu 1.5, Phe 1.99, Lys 3.30, His 1.21, Arg 3.94, Pro 1.22 (mg/g). The polysaccharides to peptides ratio in GLPP is approximately 95:5 [55].

**Table 3:** Function, mechanism and origin of bioactive components of *Ganoderma lucidum* with anti-aging or anti-aging related properties.

Function	Mechanism	Bioactive components	Origin	References
Lifespan extension	Binding to TIR-1 and activating the rab-1/pmk-1 signaling pathway to induce the expression of DAF-2	RF3	Fruit body	[61]
Antioxidant activity	Increase hydroxyl and DPPH radical scavenging activities as well as metal chelating activity	*G. lucidum* polysaccharides I, II, III, IV	Mycelia	[64-66]
	Increase scavenging of hydroxyl radicals, reactions with free oxygen species or ROOH and increase metal chelating activity	GLP	Fruit body	[67, 69]
	Increase the production of NADPH, SOD, Mn-SOD, CAT, GSH and GSH-Px; protect the mitochondria in macrophages against t-BOOH induced injury; increase the oxidation of LDL	GLPP	Fruit body	[70-72]
	Induce the productions of SOD, CAT, GPx and GSH and inhibit protein and lipid peroxidation	Total *G. lucidum* triterpenes	Fruit body	[73]
Immunomodulatory effect	Increase the production of IL-1, IL-2 and IFN-γ; increase the numbers of CD14^+^CD26^+^ monocyte/macrophage, CD83^+^CD1a^+^ dendritic cells and CD16^+^CD56^+^ NK cells; increase the cytotoxicity of CD56^+^ NK cells	RF3	Fruit body	[74, 75]
	Increase the proliferation of macrophages and their activation through increase in the production of NO	*G. lucidum* polysaccharides I, II, III, IV	Mycelia	[64]
	Activate NF-κB pathway to decrease the production of IL-8 and MCP-1	GLPP	Fruit body	[76]
	Inhibit the production of TNF-a, INF-γ and the secretion of IL-17a	GAC1	Fruit body	[59, 77]
Promotion of stem/progenitor cell survival	Increase the expression of CAM, IL-1, MCP-1, MIP-1, RANTES; Increase the secretion of BMP-2, IL-11 and aggrecan; Boost TPO- and GM-CSF-like functions	RF3	Fruit body	[81]

TIR-1: toll-interleukin 1 receptor intracellular domain, DPPH: 2,2-diphenyl-1-picrylhydrazil, ROOH: hydroperoxide, NADPH: (Reduced) Nicotinamide Adenine Dinucleotide Phosphate, SOD: superoxide dismutase, Mn-SOD: manganesesuperoxidedismutase, CAT: catalase, GSH: glutathione, GSH-Px: glutathione peroxidase.

**Figure 3.**
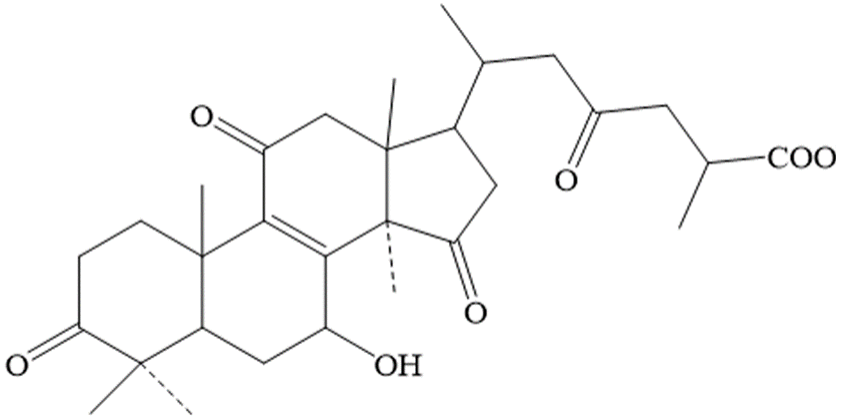
The structure of Ganoderic acid C1 (GAC1).

*Ganoderma lucidum* triterpene is another major bioactive constituent of *Ganoderma lucidum* besides polysaccharides. More than 140 distinct triterpenes have been extracted from *Ganoderma lucidum*. They have been reported to be equipped with anti-oxidation, anti-radiation, anti-tumor and immunomodulatory effects. There are no reports directly linking the anti-aging effect of *Ganoderma lucidum* triterpene, but its antioxidant property suggests that *Ganoderma lucidum* triterpene would have a potential lifespan extension effect. The anti-tumor effect of *Ganoderma lucidum* triterpenes has been shown in many different types of tumors, such as breast [56], lung [57], and cervical cancers [58]. The triterpene prodect in *Ganoderma lucidum* associated with anti-aging are total triterpene and Ganoderic acid C1. Total triterpenes are isolated from the fruit bodies of *Ganoderma lucidum*. Dissolving the ethanol extract of *Ganoderma lucidum* fruit bodies in chloroform and allows one to collect the soluble fraction. It is then concentrated and eluted with petroleum ether, chloroform, methanol, or the combination of these solvents. Fractions are finally screened for characteristics of triterpene to obtain total triterpenes of *Ganoderma lucidum*. Ganoderic acid C1 (GAC1) is a triterpenoid isolated from *G. lucidum*. It has been suggested that GAC1 is the most potent triterpenoid of *Ganoderma lucidum*, which could inhibit the release of cytokines to the similar extent as *G. lucidum* polysaccharides to modulate the immune cascade. The chemical structure of GAC1 is shown in Figure 3 [59]. The function, mechanism and origin of several anti-aging or anti-aging related components in *Ganoderma lucidum* are summarized in Table 3.

### 3.1 Lifespan extension by bioactive components from *Ganoderma lucidum*

Among the bioactive components from *Ganoderma lucidum*, only RF3 has been reported to possess the ability to extend lifespan. RF3 could bind to a membrane bound receptor to increase the expression of Toll-interleukin 1 receptor intracellular domain (TIR-1). TIR-1 is associated with aging and innate immunity in *Caenorhabditis elegans* [60], so that RF3 exerts its anti-aging effect mainly by upregulating TIR-1. Furthermore, RF3 could bind to an uncertain membrane surface receptor to activate the MAPK signaling pathway thereby increasing the transcription of rab-1/pmk-1, which induces the expression of lifespan and longevity-related transcription factor DAF-16 to subsequently extend lifespan. Since TIR-1 could also inhibit rab-1transcription, it is supposed that RF3 may upregulate TIR-1 and RAB-1 through different mechanism in the process of lifespan elongation. Acetic acid could inhibit the expression of the trans-membrane receptor DAF-2 and indirectly increase the level of DAF-16 to induce lifespan extension in *Caenorhabditis elegans*. Therefore, the combination of acetic acid and RF3 could strengthen the anti-aging effect of RF3 by 30-40% compared with using RF3 alone [61].

### 3.2 Anti-aging related effects of bioactive components from *Ganoderma lucidum*

#### 3.2.1 Antioxidant activity

Evidence has shown that *Ganoderma lucidum* polysaccharides GLP-I, GLP-II, GLP-III, GLP-IV, *Ganoderma lucidum* total triterpenes, *Ganoderma lucidum* peptide and *Ganoderma* polysaccharide peptide possess antioxidant properties. The antioxidant enzymes and GSH help protect cells against oxidative stress. SOD, CAT, and GPx aid in the clearing of ROS, while GSH protects against protein oxidation. Under oxidative stress conditions, the hydroxyl radicals could cross the cell membranes and react with almost all the intracellular molecules leading to cellular functional disorder and finally cause cell death [62].

The antioxidant activity of GLP-I, GLP-II, GLP-III and GLP-IV are measured *in vitro* by hydroxyl radical scavenging activity, metal chelating activity, DPPH radical scavenging activity, SOD-like activity assay and reducing powder test. It is proven that GLP-I, GLP-II, GLP-III and GLP-IV have differing levels of hydroxyl radical scavenging: GLP-III > GLP-IV > GLPI > GLP-II [63-64]. Transition metals could act as catalysts to promote the production of radicals and finally lead to radical-mediated oxidative chain reactions. GLP-I, GLP-II, GLP-III and GLP-IV also possess metal chelating activities, which could decrease the amount of transition metals available. GLP-III and GLP-IV have stronger metal chelating capability *vs.* GLP-I and GLP-II. Two of the four polysaccharides have strong scavenging activities with respect to the DPPH radical, in a concentration-dependent manner. The DPPH scavenging activity of GLP-IV is the strongest, followed by GLP-III, GLP-II and GLP-I. Further, the 2,2’-azinobis (3-ethylbenzothiazolin-6-sulphonic acid) (ABTS) assay can be used to evaluate the total antioxidant power of a substance [65] with GLP-IV possessing the strongest ABTS scavenging ability followed by GLP-III, GLP-I and GLP-II. These polysaccharides also have SOD-like activities and act as strong reducing agents [66].

*Ganoderma lucidum* peptide (GLP) mainly participates in decreasing lipid peroxidation partly through scavenging ^?^OH [67]; ^?^OH is the major active ROS involved in lipid oxidation [68]. GLP stops the chain reaction involved in lipid metabolism by reacting with free ^?^OH to decrease the degree of lipid peroxidation and regain mitochondrial homeostasis [69]. Furthermore, GLP eliminates free radical overloading and chelates metals to reduce metal-induced oxidation.

As the binding of *Ganoderma* polysaccharides to peptide, *Ganoderma* polysaccharide peptide (GLPP) could decrease the level of oxidative enzyme NADPH, NADPH-dependent ROS production and attenuate the MDA level in a model of renal ischemia reperfusion, while simultaneously increasing antioxidative enzymes SOD, Mn-SOD, CAT, GSH and GSH-Px. Moreover, it protects the mitochondria in macrophages against tert-butyl hydroperoxide (t-BOOH)-induced injury [69], improve mitochondria dysfunction and attenuate apoptosis induced by mitochondrial stress [70]. Furthermore, GLPP could decrease low density lipoprotein (LDL) oxidation by decreasing its relative electrophoretic mobility and the level of ox-LDL induced by CuCl_2_ [71].

*Ganoderma lucidum* triterpenes were shown to increase the levels of SOD, CAT, GPx and GSH in liver and brain tissues [72]. Administration of *G. lucidum* triterpenes could protect body against oxidative-stress-induced protein and lipid peroxidation [72].

#### 3.2.2 Immunomodulatory effect

The immunomodulatory effect of the bioactive components in *Ganoderma lucidum* includes stimulating proliferation of immune cells and cytokine expression. RF3, GLP-I, GLP-II, GLP-III, GLP-IV, GAC1 have been reported to possess immunomodulatory effects independent of any disease circumstances, while the immunomodulatory effect of GLPP is seen in effectively treating rheumatoid arthritis.

RF3 exerts its immunomodulatory effect through the stimulation of mice spleen cell proliferation and cytokine (especially IL-1, IL-2 and IFN-γ) expression [73]. RF3 could also regulate immunophenotypic expression in mononuclear cells. Treating mononuclear cells with RF3 increased the populations of CD14^+^CD26^+^ monocyte/macrophage, CD83^+^CD1a^+^ dendritic cells and CD16^+^CD56^+^ NK-cells. Furthermore, NK-cells in UCB play an important role in immune surveillance against cancer and the use of RF3 in umbilical cord blood (UCB) could significantly increase the cytotoxicity of CD56^+^ NK-cells [74].

GLP-I, GLP-II, GLP-III and GLP-IV could stimulate macrophage proliferation and induce NO production in macrophages. NO is a highly reactive free radical, which is involved in the formation of oxidation products. Since NO is significant in the activation of non-specific host defense, it can be used to measure macrophage activation. The relative potential of GLPs on NO production is ranked as follows GLP-IV > GLP-III > GLP-II > GLP-I [64].

GLPP’s immunomodulatory is seen in its effectiveness in treating rheumatoid arthritis. IL-8 and MCP-1 are key inducers of rheumatoid arthritis. The downregulation of fibroblastic IL-8 and MCP-1 could attenuate leukocyte aggregation and activation, and decrease chemokine production [75]. GLPP could activate the NF-κB signal transduction pathway to decrease IL-8 and MCP-1 production thereby exerting its anti-inflammation and immunomodulatory effects.

GAC1 was demonstrated to inhibit the release of TNF-α in LPS-stimulated murine macrophages and asthma patients’ peripheral blood mononuclear cells (PBMNCs). In asthma, GAC1 inhibits TNF-α secretion through the NF-κB pathway [59]. Besides asthma, GAC1 also activates macrophages and decreases the secretion of inflammatory cytokines including TNF-α, IFN-γ, and IL-17α in PBMNCs and a colonic biopsy of patients with Crohn’s disease [76]. Excessive TNF-α production could directly mediate or exacerbate several inflammatory diseases including Crohn’s disease [77], rheumatoid arthritis [78], and asthma [79]. A series of anti-TNF-α medications are currently used to treat inflammatory diseases. The inhibition of TNF-α secretion by GAC1 is of importance with regards to its immunomodulatory effect.

### 3.3 Promotion of stem/progenitor cell surviva

Stem/progenitor cells can replace damaged and senescent cells and participate in maintaining body homeostasis. Therefore, preserving or enhancing the survival of stem/progenitor cells could induce an anti-aging effect. Agents with the function of preserving stem/progenitor cells have been used in the field of regenerative medicine and anti-aging. RF3 was found to promote the survival of hematopoietic stem/progenitor cells [80]. RF3 exhibits cytokine- and chemokine-like functions, involving increasing the expression of CAM (N-CAM, I-CAM), IL-1, MCP-1, MIP-1, RANTES as well as the secretion of autokines (BMP-2, IL-11). It also possesses thrombopoietin (TPO)- and GM-CSF-like functions [80].

## 4. Extractions or bioactive components with potential anti-aging effects from the other species of *Ganoderma*

*Ganoderma* is a big family of herbal medicine. Some other *Ganoderma* species not only *Ganoderma lucidum, including Ganoderma neo-japonicum*, *Ganoderma tsugae and Ganoderma atrum*, have also been demonstrated to have potential anti-aging effects. Their origin, function and mechanisms are summarized in Table 4.

### 4.1 Aqueous extracts of *Ganoderma lucidum* and *Ganoderma neo-japonicum*

Neuronal senescence is a core reason of age-related neurodegeneration. The aqueous extracts of *G. lucidum* and *G. neo-japonicum* possess potential anti-aging properties through their promotion of neuritogenesis [81]. Comparing with *G. lucidum*, *G. neo-japonicum* is more effective in inducing neuritogenesis in PC12 cells. They induce this effect by mimicking NGF, which promotes neuronal survival and neuritogenesis [82]. Furthermore, the aqueous extracts of *G. lucidum* and *G. neo-japonicum* induce neuroprotection through the MEK/ERK1/2 and PI3K/Akt signaling pathways [81].

**Table 4:** The origin, function and mechanisms of extractions or bioactive components from other *Ganoderma* species exerting potential anti-aging effects.

Bioactive components	Origin	Function	Mechanism	Refs.
Aqueous extracts of *G lucidum* and *G. neo-japonicum*	Fruit bodies of *G lucidum* and *G. neo-japonicum*	Neuroprotection	Promote neuritogenesis through the MEK/ERK1/2 and PI3K/Akt signaling pathways	[82, 83]
Methanolic extract of *G. lucidum, G. lucidum antler* and *G. tsugae*	Fruit bodies of *G. lucidum, G. lucidum antler* and *G. tsugae*	Antioxidant activity	Strong DPPH scavenging effect and ferrous ion chelating activity	[84]
*G. atrum polysaccharide (PSG-1)*	Fruit body of *G. atrum*	Lifespan extension	Decrease oxidative stress in aged mice; Relieve immune dysfunction through upregulation of serum IL-2 level and increasing lymphocyte proliferation	[86, 87]
		Antioxidant activity	Increase the production of SOD, CAT, GSH and GPx; decrease the level of MDA and ROS	[92, 93, 94]
		Immunomodulation	Induce production of IL-2 and increase activation of spleen lymphocytes through Ca^2+^/calcineurin/nuclear factor of activated T cells (NFAT) pathway or protein kinase C (PKC)/NFAT pathway; Induce the release of TNF-α during macrophage activation through the TLR4/ROS/PI3K/Akt/MAPKs/NF-κB pathway	[95, 96, 97]
Polysaccharide from submerged fermentation culturing mycelium powder of *G. capense*	Culturing mycelium powder of *G.* capense	Promotion of neuronal differentiation	Strong 1-diphenyl-2-picryl-hydrazyl (DPPH•) and hydroxyl radical-scavenging abilities	[99, 100]
		Anti-glycation activity	Inhibit the formation of advanced glycation end products	[101]

### 4.2 Methanolic extract of co-culture of *Ganoderma lucidum*, *Ganoderma lucidum* antler and *Ganoderma tsugae*

The methanolic extract of a co-culture of *G. lucidum*, *G. lucidum* antler and *G. tsugae* have documented antioxidant properties. Total phenols are the main antioxidant ingredients in the extract. The extract has a strong scavenging effect on the 1,1-diphenyl-2-picrylhydrazyl and hydroxyl radicals as well as a chelating effect on ferrous ions [83]. The extraction may have an anti-aging effect due to its antioxidant property.

### 4.3 *Ganoderma atrum* polysaccharide (PSG-1)

*G. atrum* polysaccharide (PSG-1) is a homogeneous protein-bound polysaccharide with an average molecular weight of 1013 kDa, which is the most abundant ingredient of *G.* atrum (a specialized kind of *Ganoderma*). It is purified from the fruit body of *G.* atrum with a purity of more than 99.8% [84]. PSG-1 is composed of glucose, galactose and mannose in the respective proportions of 1: 1.28: 4.91.

#### 4.3.1 Lifespan extension by PSG-1

It has been comfirmed that intraperitoneal injection of PSG-1 for 4 weeks was shown to relieve oxidative stress, immune dysfunction [85] and age-related injury in the aged mouse brain [86] in a dose dependent manner.

The increase in oxidative stress by D-Galactose (D-gal is a drug used in animals to induce immune system deficit, oxidative stress damage and neurochemical changes similar to normal aging) manifestaed as the upregulation of MDA. PSG-1 could decrease the level of MDA in the liver, brain and spleen of aged mice. H_2_O_2_ is the main inducer of oxidative damage, and normal cells are equipped with an oxidative stress buffer system, including several antioxidant enzymes such as SOD, CAT and GPx as well as some non-enzymes - GSH, α-tocopherol, vitamin C, carotene and flavonoids. SOD could convert H_2_O_2_ into O_2_^-^ and GPx or CAT could degrade H_2_O_2_ to form non-toxic products. Furthermore, the increase in the activities of SOD, CAT, GPx and GSH prevents DNA, lipid and protein damage induced by oxidative stress in the process of aging [87]. PSG-1 relieves oxidative damage and delays aging process by increasing the activities of SOD, CAT, GPx and GSH in the blood [85]. Moreover, PSG-1 exerts a protective effect by decreasing the level of ROS and thus the level of calcium accumulation [86], since the disruption of calcium homeostasis is caused by ROS accumulation [88].

Recently, a study reveals that there is a positive correlation between immune function and longevity suggesting good lymphocyte function leads to a longer lifespan. Lymphocytes play important roles in the regulation of immune responses [89] and IL-2 is involved in the regulation of lymphocyte proliferation. The aging process decreases the production of IL-2 and causes immune dysfunction [90]. PSG-1 has been shown to relieve immune dysfunction through the upregulation of serum IL-2, which increases lymphocyte proliferation and subsequently, longevity [85].

#### 4.3.2 Anti-aging related effects of PSG-1

##### 4.3.2.1 Antioxidant activity

PSG-1 has obviously anti-oxidant activity. PSG-1 could relieve diabetes through its antioxidant function. It decreases fasting blood glucose level and exerts protection on the endothelium of diabetic rats by decreasing ROS release [91] and/or the activation of the PI3K/Akt/eNOS pathway [92]. PSG-1 has also been shown to improve hepatic function through its antioxidant activity, which participates in decreasing serum aspartate aminotransferase (AST) and alanine aminotransferase (ALT) levels and increasing hepatic glycogen [93].

##### 4.3.2.2 Immunomodulation

PSG-1 could stimulate the production of IL-2 and increase the activation of spleen lymphocytes through the Ca^2+^/calcineurin (CaN) /nuclear factor of activated T cells (NFAT) pathway or protein kinase C (PKC)/NFAT pathway [94]. The release of TNF-α during macrophage activation is induced by PSG-1 through the TLR4/ROS/PI3K/Akt/MAPKs/NF-κB pathway [95]. Furthermore, PSG-1 is touted to be anti-cancerous in that it could increase phagocytic ability and production of NO and ROS in S-180 tumor cells by activating TLR-4, MAPK, Akt and NF-κB. While in CT26 tumor cells, PSG-1 exerts its anti-tumor effect mainly through the activation of cyclic AMP (cAMP)/protein kinase A (PKA) pathway, Ca^2+^/PKC pathway or mitochondria-mediated apoptotic pathway to increase the apoptotic rate of tumor cells, decrease tumor weight and increase the immune organ index to activate host immune function of tumor cell-bearing mice [96].

**Figure 4.**
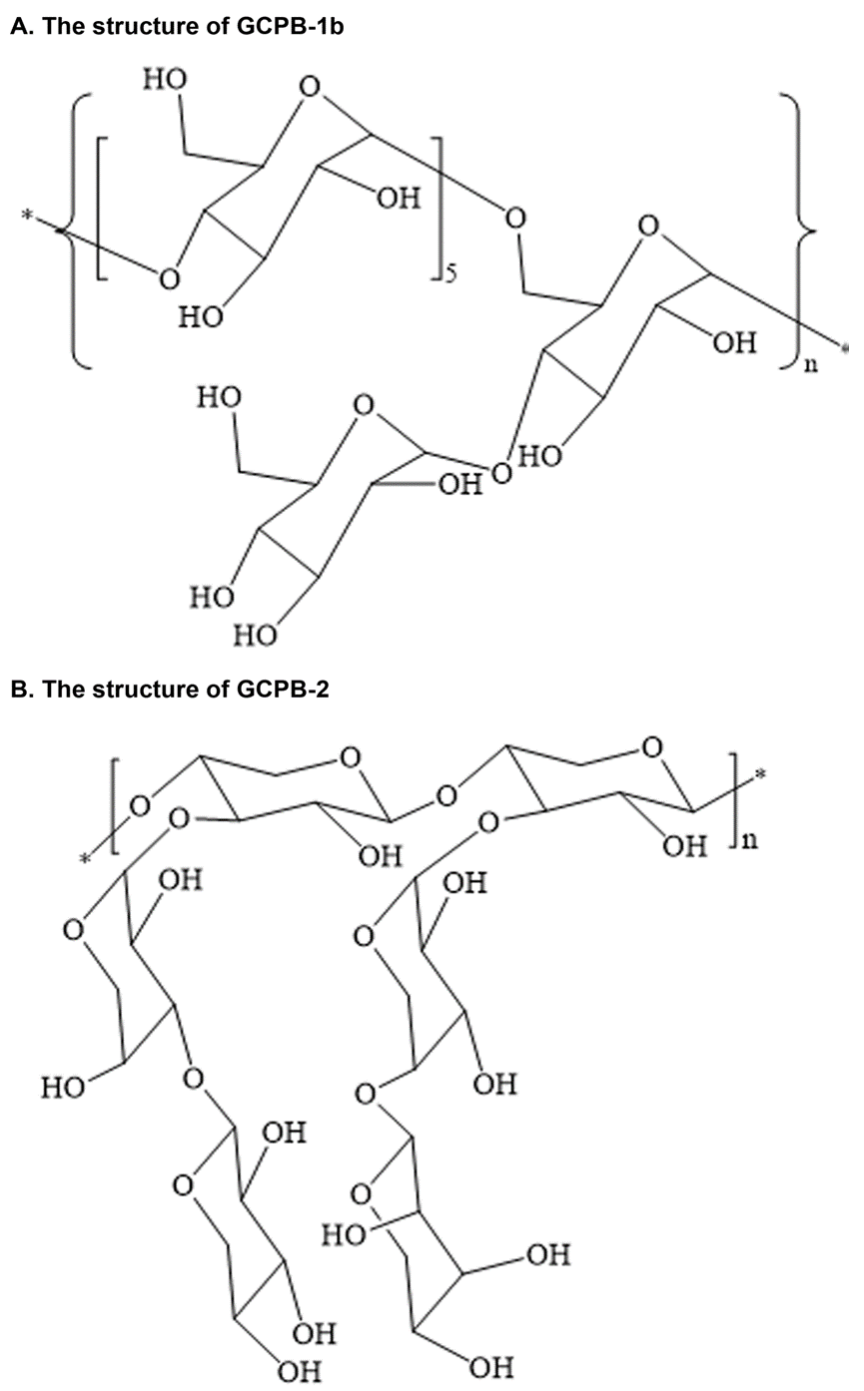
The structures of polysaccharides from submerged fermentation culturing mycelium powder of Ganoderma capense (GCPB-1b and GCPB-2) **A**) The chemical structure of GCPB-1b. **B**) The chemical structure of GCPB-2.

##### 4.3.2.3 Modifications and derivatives of PSG-1

Considering its strong antioxidant and immunomo-dulatory effects, scientists are wondering if structural modifications to PSG-1 could increase its advantages and promote its clinical application. It turns out that the modifications of PSG-1 have stronger immunomo-dulation and antioxidant abilities than native PSG-1. Sulfated polysaccharides (S-PSG) and the acetylated or carboxymethylated derivatives of PSG-1 have higher macrophage phagocytosis and TNF-α production capacity as well as stronger DPPH radical scavenging ability compared with its native form [97].

### 4.4 Polysaccharide from submerged fermentation culturing mycelium powder of Ganoderma capense

Like its counterpart *Ganoderma lucidum*, *G. capense* is also a well-known medicinal mushroom that has been widely used for the treatment of several chronic diseases [98]. It has been reported recently that the polysaccharides from submerged fermentation culturing mycelium powder of *G. capense* possesses an anti-aging effect. These polysaccharides include GCPB-1b, GCPB-2, GC50, GC70, GC90 and GCB. The structures of GCPB-1b and GCPB-2 are shown in Figure 4. Both GCPB-1b and GCPB-2 have strong DPPH• scavenging abilities [98-99].

The *in vitro* antiradical examination shows that GC50, GC70, GC90 and GCB have concentration-dependent DPPH and hydroxyl scavenging abilities. The evaluation of anti-glycation activity using a non-enzymatic glycation reaction indicates that GC70 could inhibit the formation of advanced glycation end products [100]. We posit that GCPB-1b, GCPB-2, GC50, GC70, GC90 and GCB are likely to possess direct anti-aging effects, since the antioxidant and anti-glycation effects of the six polysaccharides were tested in the *in vitro* system other than *in vivo*. Further studies are required in revealing whether they have lifespan elongation effect.

## 5. Conclusion

In conclusion, a variety of *Ganoderma lucidum* extracts have definite anti-aging properties and they exert their anti-aging effects mainly through anti-oxidation, immunomodulation and anti-neurodegeneration. *G. lucidum* has been viewed as an elixir since Chinese ancient times. Apart from its longevity effects, many studies have tried to demonstrate the other healthy promoting properties such as anti-diabetes and anti-cancer based on its anti-senescence effect. Therefore, it is of paramount importance to obtain a full picture of all the anti-aging ingredients and extracts in *G. lucidum*. Additional evidence is required to provide a comprehensive explanation of the mechanisms underlying the anti-aging property of* G. lucidum*.
